# The effect of short-term canola oil ingestion on oxidative stress in the vasculature of stroke-prone spontaneously hypertensive rats

**DOI:** 10.1186/1476-511X-10-180

**Published:** 2011-10-17

**Authors:** Annateresa Papazzo, Xavier Conlan, Louise Lexis, Paul Lewandowski

**Affiliations:** 1 School of Medicine, Deakin University, Victoria, Australia; 2 Institute for Technology Research and Innovation, Deakin University, Victoria, Australia; 3 Department of Human Biosciences, La Trobe University, Victoria, Australia

**Keywords:** canola oil, SHRSP rats, superoxide dismutase, NADPH oxidase, oxidative stress

## Abstract

**Background:**

This study aimed to determine if 25 days of canola oil intake in the absence of excess dietary salt or together with salt loading affects antioxidant and oxidative stress markers in the circulation. A further aim was to determine the mRNA expression of NADPH oxidase subunits and superoxide dismutase (SOD) isoforms in the aorta of stroke-prone spontaneously hypertensive (SHRSP) rats.

**Methods:**

Male SHRSP rats, were fed a defatted control diet containing 10% wt/wt soybean oil or a defatted treatment diet containing 10% wt/wt canola oil, and given tap water or water containing 1% NaCl. Blood was collected at the end of study for analysis of red blood cell (RBC) antioxidant enzymes, RBC and plasma malondialdehyde (MDA), plasma 8-isoprostane and plasma lipids. The aorta was removed and the mRNA expression of NOX2, p22*^phox^*, CuZn-SOD, Mn-SOD and EC-SOD were determined.

**Results:**

In the absence of salt, canola oil reduced RBC SOD and glutathione peroxidase, and increased total cholesterol and LDL cholesterol compared with soybean oil. RBC glutathione peroxidase activity was significantly lower in both the salt loaded groups compared to the soybean oil only group. In addition, RBC MDA and plasma HDL cholesterol were significantly higher in both the salt loaded groups compared to the no salt groups. Plasma MDA concentration was higher and LDL cholesterol concentration lower in the canola oil group loaded with salt compared to the canola oil group without salt. The mRNA expression of NADPH oxidase subunits and SOD isoforms were significantly reduced in the canola oil group with salt compared to canola oil group without salt.

**Conclusion:**

In conclusion, these results indicate that canola oil reduces antioxidant status and increases plasma lipids, which are risk factors for cardiovascular disease. However, canola oil in combination with salt intake increased MDA, a marker of lipid peroxidation and decreased NAPDH oxidase subunits and aortic SOD gene expression.

## Background

Evidence has shown that ingestion of canola oil as the sole dietary fat source (added at 10% wt/wt to standard rat chow) shortens the life span of stroke-prone spontaneously hypertensive (SHRSP) rats compared to the soybean oil or perilla oil [1-7]. Our recent study strengthened this finding, and showed that canola oil ingestion reduced the lifespan of SHRSP rats compared to soybean oil following 1% NaCl loading, 85.8 ± 1.1 and 98.3 ± 3.4 days, respectively [8].

The mechanism by which canola oil reduces life span is currently unknown; however, decreased antioxidant activity and heightened oxidative stress have been implicated. Our previous study showed that canola oil intake reduced the antioxidant activities of red blood cell (RBC) superoxide dismutase (SOD), glutathione peroxidase (GPx) and catalase compared to soybean oil in SHRSP rats following NaCl loading at the end of their life span [8]. Furthermore, canola oil intake increased plasma MDA compared to pre-treatment, suggesting an increase in lipid peroxidation overtime [8]. RBCs can provide protective mechanisms against oxidative damage to endothelial cells by neutralising reactive oxygen species (ROS) in the circulation [9]. Previous research has shown an inverse relationship between reduced activities of antioxidants (SOD and GPx) and increased lipid peroxidation products in blood and cardiovascular disease (CVD) [10]. Evidence has shown that in canola oil fed spontaneously hypertensive rats (SHR) there was an increase in RBC glutathione and glutathione reductase, with a decrease in the activity of RBC GPx. Furthermore, in the hepatic cytosol, the activity of SOD and catalase were significantly reduced [11]. Similar results were also found in a study by Ohara et al. [12], in which the activities of catalase, GPx and glutathione reductase were decreased in the liver of canola oil fed Wistar-Kyoto (WKY) rats. Taken together these results indicate that canola oil ingestion affects antioxidant enzyme activity in different tissues.

In vascular cells, nicotinamide adenine dinucleotide phosphate (NADPH) oxidase is a major source of ROS, and is functionally active within all the layers of the vessel wall [13-15]. In hypertensive patients, vascular smooth muscle cells (VSMCs) from resistance arteries have increased ROS generation, and this increase is linked to NADPH oxidase [16]. Evidence has shown that in SHR and SHRSP rats there was an enhanced production of superoxide (·O_2_ˉ) derived from NADPH oxidase, and this was associated with the upregulation of p22^phox ^mRNA expression in the aorta [14,17]. Furthermore, NOX2 mRNA expression in the aorta was found to be greater in SHR compared to the normotensive WKY rats [18]. In vascular cells, SOD is a major cellular antioxidant that provides defence against ·O_2_ˉ [15]. There are three isoforms of SOD which have been identified and include a cytosolic copper/zinc-containing SOD (CuZn-SOD), mitochondrial SOD (Mn-SOD), and extracellular SOD (Ec-SOD). The main vascular SOD is Ec-SOD and is produced and secreted by VSMCs [15,16]. Evidence has shown that in atherosclerotic vessels Ec-SOD expression is increased in apoE-deficient mice, a mouse model of atherosclerosis [19]. The increase in Ec-SOD expression may be an adaptive response to an increase in oxidative stress [19].

Furthermore, the concentration of phytosterols in canola oil has also been suggested to be a contributing factor to the shorten life span. However, conflicting results have shown no clear correlation between the content of phytosterols in the diet and tissues and survival time observed [5,20]. Moreover, NaCl loading may be masking the effects of dietary phytosterols and canola oil in the SHRSP rat. A recent study showed that dietary phytosterols and phytostanols increase blood pressure in Wistar Kyoto rats in the absence of NaCl loading [21]. Furthermore, research has shown that salt intake can induce oxidative stress, and leads to an increase in ·O_2_ˉ production in SHR and Sprague-Dawley rats [22,23]. More research is required to investigate the effect of canola oil intake on oxidative damage and to examine the changes in the absence of excess dietary salt. This study aimed to determine if 25 days of canola oil intake in the absence or together with salt loading effects the antioxidant and oxidative stress markers in circulation and mRNA expression of NADPH oxidase subunits and SOD isoforms in the aorta of SHRSP rats.

## Results

### Body weight, food intake and water intake

**Figure 1:**
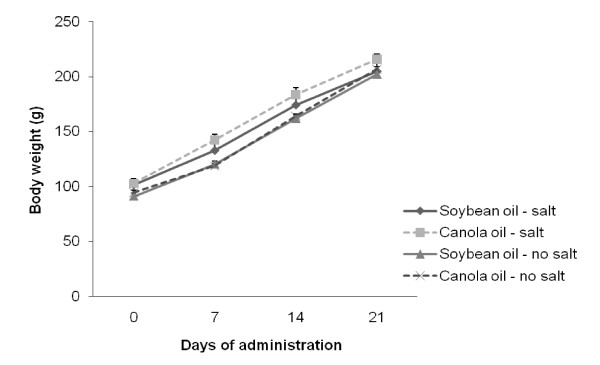
**Mean body weight of SHRSP rats fed canola oil compared with soybean oil diet in the absence or presence of NaCl loading**. Vaules are means ± SEM.

**Figure 2:**
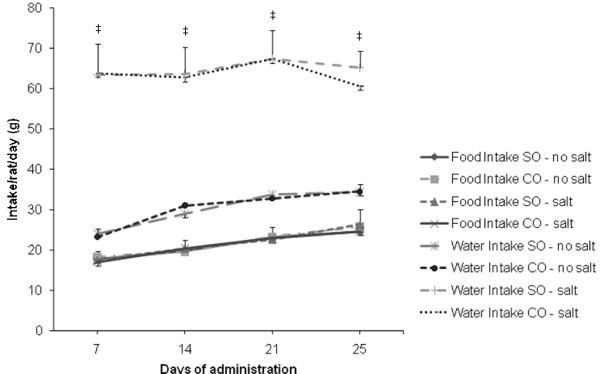
**Food and water intake of SHRSP rats fed canola oil compared with soybean oil diet in the absence or presence of NaCl loading**. Values are means ± SEM. ^‡^*P *< 0.05 represents a significant difference from soybean oil and canola oil no salt groups.

Body weight of the animals increased gradually over the course of the trial in all diet groups. There were no significant differences between soybean oil and canola oil groups (Figure 1). There were also no significant differences in food consumption between all dietary groups. The water intake in soybean oil and canola oil groups with salt were significantly increased (*P *< 0.05) compared to the soybean oil and canola oil groups without salt (Figure 2).

### Blood pressure

**Figure 3:**
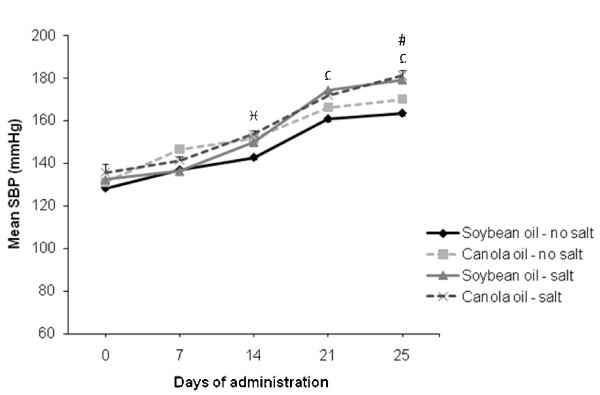
**Mean systolic blood pressure of SHRSP rats fed canola oil compared with soybean oil in the absence or presence of salt**. Vaules are means ± SEM. ^Ω^*P *< 0.05 represents a significant difference between soybean oil no salt and soybean oil with salt groups; ^^*P *< 0.05 represents a significant difference between canola oil no salt and soybean oil no salt groups; ^#^*P *< 0.05 represents a significant difference between canola oil no salt and canola oil with salt groups.

Blood pressure increased over time in all diet groups, with significant changes (*P *< 0.05) seen between different groups at days 14, 21 and 25 (Figure 3). At day 14, the blood pressure was significantly increased (*P *< 0.05) in the canola oil group without salt compared to the soybean oil group without salt. At day 21 and 25, the blood pressure was significantly increased (*P *< 0.05) in the soybean oil group with salt compared to the soybean oil group without salt. Also, at day 25 the blood pressure was significantly increased (*P *< 0.05) in the canola oil group with salt compared to the canola oil group without salt.

### Antioxidant enzymes and oxidative damage

Markers of antioxidant status and oxidative damage are represented in Table 1. In the absence of salt, canola oil ingestion significantly reduced (*P *< 0.05) the activities of RBC SOD and GPx compared with soybean oil alone. The activity of RBC GPx was significantly reduced (*P *< 0.05) in the soybean oil and canola oil groups in the presence of salt compared with soybean oil alone. There were no significantly differences in the activity of catalase between the groups.

**Table 1:** Antioxidant status and oxidative damage in SHRSP rats fed canola oil compared with soybean oil diets in the absence and presence of salt

	Soybean oil no salt	Canola oil no salt	Soybean oil salt	Canola oil Salt
RBC SOD (U/gm Hb)	484.7 ± 76.7	277.6 ± 51.9*	337 ± 68.1	354.9 ± 59.9
RBC GPx (mmol/min/gm Hb)	85.4 ± 4.1	57.2 ± 9*	62.4 ± 8.7*	61.4 ± 7.8*
RBC Catalase (mmol/min/gm Hb)	377.6 ± 40.1	320.4 ± 36.6	303.2 ± 33.8	331.1 ± 42.1
RBC MDA (μM)	10.2 ± 0.3	10.2 ± 0.3	11.1 ± 0.2^‡^	11.1 ± 0.2^‡^
Plasma MDA (μM)	15.9 ± 0.6	14.5 ± 0.4	15.8 ± 1.1	16.4 ± 0.5^#^
Plasma 8-isoprostane (pg/ml)	64.9 ± 8.5	83.2 ± 10.3	86.6 ± 8.7	105.1 ± 15.5

Values are means ± SEM; SOD, superoxide dismutase; GPx, glutathione peroxidase; MDA, malondialdehyde. *P < 0.05 represents a significant difference from soybean oil no salt group; ^#^P < 0.05 represents a significant difference from canola oil no salt group; ^‡^P < 0.05 represents a significant difference from soybean oil and canola oil no salt groups.

Canola oil and soybean oil ingestion with salt loading significantly increased (*P *< 0.05) RBC MDA compared to both the canola oil and soybean oil groups without salt. Plasma MDA in the canola oil group in the presence of salt was significantly higher (*P *< 0.05) than the canola oil group without salt. There were no significantly differences in the concentration of 8-isoprostane between the groups.

### Plasma lipids

Canola oil ingestion alone significantly increased (*P *< 0.05) the concentration of total cholesterol and LDL-C compared to soybean oil alone. LDL-C concentration was significantly lower (*P *< 0.05) in the canola oil group loaded with salt compared to the canola oil group without salt. HDL-C concentration was significantly higher (*P *< 0.05) in both the salt loaded groups compared to the no salt groups. There were no significant differences in the concentration of triglycerides between groups (Table 2).

**Table 2:** Plasma lipids in SHRSP rats fed canola oil compared with soybean oil diets in the absence and presence of salt

	Soybean oil no salt	Canola oil no salt	Soybean oil salt	Canola oil salt
Total cholesterol (mmol/L)	3.1 ± 0.1	3.4 ± 0.1*	3.1 ± 0.1	3.3 ± 0.1*
LDL-C (mmol/L)	1.4 ± 0.1	1.7 ± 0.1*	2.2 ± 1.3	1.1 ± 0.2^#^
HDL-C (mmol/L)	1.3 ± 0.1	1.4 ± 0.1	2.3 ± 1.1^‡^	2.3 ± 0.4^‡^
Triglycerides (mmol/L)	1.8 ± 0.1	2 ± 0.1	1.7 ± 0.1	1.9 ± 0.1

Values are means ± SEM; ^‡^P < 0.05 represents a significant difference from soybean oil and canola oil no salt groups; *P < 0.05 represents a significant difference from soybean oil no salt group; ^#^P < 0.05 represents a significant difference from canola oil no salt group.

### mRNA gene expression

In the absence of salt there were no changes seen in the mRNA expression of NOX2 and p22^phox ^(Table 3). However, when salt loading was used canola oil intake significantly reduced (P < 0.05) NOX2 mRNA expression compared to the soybean oil group with salt and the canola oil group without salt. Canola oil intake with salt significantly reduced (P < 0.05) p22^phox ^mRNA expression compared to the canola oil group without salt.

**Table 3:** Effect of canola oil intake compared to soybean oil intake on mRNA expression in the aorta of SHRSP rats in the absence and presence of salt

Gene	mRNA expression (Arbitrary units)			
	
	Soybean oil no salt	Canola oil no salt	Soybean oil salt	Canola oil Salt
p22*^phox^*	7.1 ± 2.4	7.6 ± 2.0	4.2 ± 1.4	2.6 ± 1.0^#^
NOX2	5.3 ± 1.5	9.9 ± 3.0	9.1 ± 3.6	1.9 ± 0.7^†; #^
CuZn-SOD	46.6 ± 19.4	29.7 ± 7.2	22.9 ± 4.5	8.9 ± 2.5^†; ‡^
Mn-SOD	126.1 ± 41.2	94.6 ± 20.1	93.5 ± 20.9	37.3 ± 6.7^†; ‡^
Ec-SOD	68.9 ± 17.4	82.7 ± 12.1	89.3 ± 17.2	25 ± 1.9^†; #^

Values are mean ± SEM. ^†^P < 0.05 represents a significant difference between canola oil and soybean oil with salt groups; ^#^*P *< 0.05 represents a significant difference from canola oil no salt group; ^‡^P < 0.05 represents a significant difference from soybean oil and canola oil no salt groups.

In the absence of salt, there were no changes seen in the mRNA expression of CuZn-SOD, Mn-SOD and EC-SOD (Table 3). However, when salt loading was used canola oil intake significantly decreased (P < 0.05) CuZn-SOD and Mn-SOD mRNA expression compared with soybean oil with salt group and both the canola oil and soybean oil groups without salt. EC-SOD mRNA expression was significantly reduced (P < 0.05) in the canola oil group with salt compared with soybean oil group with salt and the canola oil group without salt.

## Discussion

The data from this study have shown that canola oil ingestion alone decreased the activities of SOD and GPx compared with soybean oil by 53% and 33%, respectively. These results indicate that canola oil ingestion alone for 25 days affects antioxidant enzyme activity. Previous studies have shown that the in the canola oil feeding (with no added salt) reduced the activities of SOD and catalase in the hepatic cytosol of SHR and WKY rats [11,24]. The present study has also shown that in the presence of salt, both canola oil and soybean oil ingestion reduced the activity of GPx compared with the soybean oil group without salt. The reduced activity of RBC GPx in the salt loaded canola oil group is consistent with our previous findings [8]. However, our previous findings showed that canola oil ingestion along with salt loading in SHRSP rats reduced RBC SOD, catalase as well as GPx at the end of their mean life span, 85 days [8]. Supporting evidence shows that there is an association between reduced antioxidants and CVD [10]. In addition, previous research has shown an inverse relationship between erythrocyte GPx activity and the incidence of CVD [25].

The present study has also shown that canola oil ingestion with salt loading increased plasma MDA concentration when compared to the canola oil group without salt. In addition, RBC MDA concentration was increased in the canola oil and soybean oil groups in the presence of salt compared with the non salt groups. The increased plasma and RBC MDA concentration indicates an increased amount of ROS induced lipid peroxidation, which may be due to the salt loading in combination with the diets. A previous study found an increase in urine MDA concentration in Sprague-Dawley rats as a result of salt intake [23]. They found a difference between the low salt (0.03%) and normal salt (0.3) groups, and between the low salt and high salt (6%) groups. In addition, they also found an increase in urine 8-isoprostane levels as a result of salt intake. However, there were no changes found in the 8-isoprostane levels in the present study. In a previous study in WKY rats, canola oil ingestion in the absence of extra dietary salt increased lipid peroxide levels in the hepatic cytosol [12], while an earlier study showed no change in lipid peroxide levels in the hepatic cytosol of SHR with 1% NaCl loading [11]. The mechanism by which canola oil intake in combination with salt increases MDA is currently unknown.

The present study has shown that in the presence of salt, canola oil intake decreased the mRNA expression of CuZn-SOD, Mn-SOD and Ec-SOD. The decrease in the SOD isoforms indicates a reduced ability to eliminate ·O_2_ˉ in the presence of canola oil and salt. High salt intake (6%) has been shown to reduce renal expression of CuZn-SOD and Mn-SOD in Sprague-Dawley rats [23]. The present study has also showed that p22*^phox ^*and NOX2 mRNA expression was reduced in the canola oil group with the presence of salt, indicating that ·O_2_ˉ generated from NADPH oxidase may be decreased. Our current study shows for the first time that canola oil intake with salt reduces NADPH oxidase subunits and SOD isoforms in the aorta of SHRSP rats after 25 days of feeding. Evidence has shown that ·O_2_ˉ generated from NAPDP oxidase is increased in hypertension [16]. In addition, Kitiyakara et al. found that salt intake in Sprague-Dawley rats lead to an increase in ·O_2_ˉ production [23]. This was accompanied by an increase in renal activity and mRNA expression of NOX2 and p47*^phox^*, and a decrease in CuZn-SOD and MnSOD mRNA expression [23]. However, in the vasculature, there are several other sources of ROS, which include: xanthine oxidase, uncoupled nitric oxide synthase, lipoxygenase and the mitochondrial respiratory chain [26,27]. In the present study, ROS generation may be coming from other sources within the vasculature, and requires further investigation. Furthermore, it would have been ideal to examine the protein levels of the genes of interest. A study found that plasma Ec-SOD activity was decreased in hypertensive patients, while there were no changes found in protein levels [28]. Therefore, the reduction in Ec-SOD activity may not due to the down regulation of Ec-SOD. In the present study, SOD activity was reduced in the canola oil group without salt, while there were no changes seen in the mRNA expression of the SOD isoforms.

Previous studies have reported an increase in plasma lipids due to canola oil ingestion [11,12,24]. In the present study, canola oil ingestion alone increased the concentration of total cholesterol and LDL-C compared with soybean oil alone. When salt loading was used canola oil intake increased total cholesterol compared with canola oil without salt. HDL-C in both the canola oil and soybean oil groups with salt was higher compared with the non salt groups. Previous studies in SHR and WKY rats have shown increases in total cholesterol and HDL-C with administration of canola oil compared to soybean oil [11,12,24]. Research has found an association between oxidised LDL-C and the pathogenesis of atherosclerosis [29]. In the present study, the combination of canola oil and salt intake resulted in a decrease in LDL-C compared with the canola oil without salt group. This is consistent with our previous findings showing a decrease in LDL-C, as well as HDL-C and total cholesterol in the canola oil group [8]. There is substantial evidence suggesting that high salt intake increases the risk of CVD [30]. However, some studies suggest that low salt intake and its adverse effects on blood lipids can have a detrimental effect on CVD risk [30]. The mechanisms by which salt intake affects the blood lipids are not clear. A meta-analysis on humans reported that a reduction in salt intake from 20 to 200 mmol/day resulted in a significant increase in total cholesterol and LDL-C [31]. A study by Harsha et al. found that within each diet (the typical American diet (control) and the Dietary Approaches to Stop Hypertension (DASH) diet), sodium intake (50, 100 or 150 mmol/d) did not significantly affect the serum levels of LDL-C, total cholesterol, HDL-C and triglycerides [30]. However, at each sodium concentration, LDL-C, HDL-C and total cholesterol were lower in the DASH diet compared to the American control diet [30]. Taken together these results suggest that a diet low in salt leads to an increase in LDL-C.

The results of the present study show an increase in blood pressure in both the canola oil and soybean oil groups with salt compared to the dietary groups without salt at the end of the feeding trial. The association between salt intake and hypertension is well known [32], which is evident in the present the study. Evidence indicates that canola oil intake has an effect on blood pressure in the SHRSP rat and its related strains [3,24]. However, the blood pressure in the canola oil groups was not consistently different from soybean oil. Our previous study showed that blood pressure in the canola oil group was not different from soybean oil. A study by Huang et al. observed no significant change in systolic blood pressure in the canola oil group compared to the soybean oil group at 4 and 8 weeks of age [1]. Another study by Ratnayake et al. found no significant differences in the systolic blood pressure among different dietary groups in SHRSP rats [6]. Taken together these results suggest that canola oil intake in the presence or absence of salt does not affect blood pressure. Therefore, the life shortening effect of canola oil may not be directly due to an increase in blood pressure.

## Conclusions

In conclusion, canola oil ingestion in the absence of dietary salt decreased the activities of RBC SOD and GPx, and increased both total cholesterol and LDL-C, which are risk factors for CVD. However, the combination of canola oil and dietary salt intake resulted in an increase in plasma MDA, a decrease in LDL-C, and a decrease in NADPH oxidase subunits and SOD aortic expression when compared to canola oil intake alone. ROS generation may be coming from other sources within the vasculature. The increase in RBC and plasma MDA, with a decrease in RBC GPx and SOD mRNA expression may indicate an elevation in oxidative stress. More research is required to determine if canola oil intake in the absence or presence of salt leads to oxidative stress and altered vascular changes such as endothelial dysfunction in a longer duration study.

## Methods

### Animal husbandry and study design

Approval for this project was granted by the Deakin University Animal Welfare Committee (Approval no. A67/09). Forty male SHRSP rats (Deakin University, Australia) aged 4 weeks were randomly assigned to either group 1 (n = 20) or group 2 (n = 20). Within each group the rats were randomly assigned to a control or treatment group and acclimatized for one week. During acclimatization they were given a standard pellet diet (Specialty Feeds, Western Australia) and water ad libitum. The groups were then fed the following diets respectively, a defatted control diet containing 10 wt/wt% soybean oil or a defatted treatment diet containing 10 wt/wt% canola oil (Speciality Feeds, Western Australia) for 25 days. Twenty five days was chosen based on our pervious study, which showed that the mean life span of the canola oil group was 85 ± 1.1 days [8]. Given that the rats were 35 days old when they started the trial, they were on the diet for a mean of 50 days. The current study was designed to examine whether canola oil intake had an effect on oxidative stress earlier on in the rats life span and, thus, 25 days was chosen as a mid point. The fatty acid compositions of the diets are shown in Table 4. Group 1 was given water containing 1% NaCl and group 2 was given tap water throughout the trial. The reason for having the group without NaCl in the drinking water is to rule out any interfering factor the salt loading may have when analysing tissues. The animals were maintained on a 12 hr light/dark photo-period with a room temperature of 21 ± 2°C. Animal body weights, food intake and water consumption were determined once a week, while the health of the animals was monitored daily. At the end of the 25 days the rats were anaesthetised via intra-peritoneal injection with lethabarb (50 mg/kg), and blood was collected for analysis. Following this, tissue collection was carried out and the aorta was removed, washed in saline solution and snap frozen in liquid nitrogen.

**Table 4:** Fatty acid composition of canola oil and soybean oil diets

Fatty acid	Soybean oil (%)	Canola oil (%)
14:0 Myristic acid	0.2	0.1
16:0 Palmitic acid	11.0	7.0
16:1 Palmitoleic acid	0.1	0.1
18:0 Stearic acid	4.0	2.0
18:1 Oleic acid	23.0	53.0
18:2 Linoleic acid	48.0	23.0
18:3 Linolenic acid	6.0	10.0
18:4 Stearidonic acid	0	0.5
20:1 Gadoleic acid	0.2	0.1
20:5 EPA	0.2*	0.2*
22:6 DHA	0.5*	0.5*

*Fatty acid source coming from fish meal found within the diet. Information provided by Speciality Feeds (Western Australia), which produced both diets.

### Measurement of blood pressure

Blood pressure was measured weekly over the course of their life span using a tail cuff sphygmomanometer (Biopac Systems, USA). For each animal systolic blood pressure was obtained as an average of three readings as each time point.

### Blood collection and processing

After the animal was anaesthetised, blood was collected via cardiac puncture into EDTA coated tubes. Immediately after blood collection, samples were centrifuged at 600 xg for 10 minutes at 4°C. The plasma was then removed and stored at -80°C until analysis of plasma lipids: triglycerides, total cholesterol, high density lipoprotein cholesterol (HDL-C) and low density lipoprotein cholesterol (LDL-C), and MDA. RBCs were then washed 3 times by adding an equal volume of 0.9% (w/v) NaCl, mixed carefully and centrifuged at 600 xg for 10 minutes at 4°C. The supernatant was removed and discarded. An equal volume of cold distilled water and RBCs were mixed well to lyse the cells. The hemolysate was stored at - 80°C for subsequent analysis of antioxidant enzymes: SOD, catalase and GPx, and MDA.

### Erythrocyte antioxidant enzymes

SOD activity was determined using a commercially available kit (Cayman Chemical Company, USA) following the manufacturer's instructions. This assay utilizes xanthine oxidase and hypoxanthine to generate superoxide radicals that are detected by tetrazolium salt with absorbance read at 540 nm using a microplate analyser (Fusion-Alpha HT, PerkinElmer, USA). One unit of SOD is defined as the amount of enzyme required to inhibit the distmutation of the superoxide radical by 50%.

Catalase activity was determined using a commercially available kit (Cayman Chemical Company, USA) following manufacturer's instructions. This method is based on the reaction of methanol with the enzyme in the presence of an optimal concentration of hydrogen peroxide. The absorbance was read at 540 nm using a microplate analyser (Fusion-Alpha HT, PerkinElmer, USA).

GPx activity was determined using was determined using a commercially available kit (Cayman Chemical Company, USA) following manufacturer's instructions. This assay is based on the oxidation of NADPH following the reduction of hydroperoxide. A decrease in absorbance at 340 nm results from oxidation of NADPH to NADP+ and the rate of this decrease is proportional to the GPx activity in the sample. The absorbance was read at 340 nm using a microplate analyser (Fusion-Alpha HT, PerkinElmer, USA) once every minute for 10 minutes.

All erythrocyte enzyme activities were normalised to haemoglobin concentration, which was determined by adding 20 μl of 200/1 hemolysate and 480 μl of Darbkin's reagent. The sample was left to stand at room temperature for 5 minutes and the absorbance read at 540 nm using a spectrophotometer (Biochrom, UK).

### Lipid peroxidation analysis

MDA in plasma and erythrocytes was determined via high performance liquid chromatography (HPLC) according to the method by Sim et al. [33]. Briefly, 100 μl hemolysate or 50 μl plasma samples were hydrolysed with 1.3 M sodium hydroxide, incubated at 60°C for 60 minutes and cooled on ice for 5 minutes. To precipitate the proteins, 35% perchloric acid was added, cooled on ice for 5 minutes and centrifuged at 3500 xg for 10 minutes. The samples were protected from light from this step onwards. To the supernant 30 μl of 2, 3-dinitrophenylhydrazine reagent was added and incubated for 10 minutes at room temperature. The aqueous phase was extracted twice with hexane and evaporated. The dry extract was reconstituted with 100 μl mobile phase, and a 45 μl injection volume was used. MDA concentrations were determined at 310 nm using HPLC (Agilent Technologies, Australia) with an Eclipse XDB-C18 column (150 × 4.6 mm, 5 μm, 1 ml/min flow rate, 9.8 MPa backpressure). External standards (5, 10, 20 and 40 μM) of MDA aliquots of suitable concentrations were used.

Total 8-isoprostane concentrations were analysed in plasma using an enzyme immunoassay (EIA) kit (Caymen Chemical Company, USA) following manufactures instructions. Prior to analysis plasma samples were hydrolysed by addition of 25 μl 2 M NaOH to each 100 μl plasma sample. The samples were incubated at 45°C for 2 hours. Following this, 25 μl 10M HCl acid was added and the samples were centrifuged for 5 minutes at 12, 000 × *g*. The supernatant was removed and used for the determination of total 8-isoprostane using the EIA kit. This assay is based on the competition between 8-isoprostane and an 8-isoprostane acetycholinesterase (AChE) conjugate for a limited number of 8-isoprostane -specific rabbit anti-serum binding sites. Values were expresses as pg/ml of plasma.

### Plasma lipids analysis

Plasma triglycerides, total cholesterol and high-density lipoprotein cholesterol (HDL-C) were determined using commercially available kits (Thermo Electron Corporation, USA) in a 96 well plate format (Fusion-Alpha HT, PerkinElmer, USA), following manufactures instructions.

Low-density lipoprotein cholesterol (LDL-C) was determined using the Friedewald equation [34]: LDL cholesterol = Total cholesterol - HDL cholesterol - (triglycerides/5).

### Reverse transcription-real-time PCR measurement of mRNA

The gene expression of NOX2, p22^phox^, CuZn-SOD, MnSOD and Ec-SOD were determined. RNA was extracted from the aorta using TRI reagent (Molecular Research Centre, USA) following the manufactures instructions. Total RNA concentration was determined using the NanoVue (GE Healthcare, Australia) and first-strand cDNA was generated from 0.5 μg RNA using the Marligen first strand cDNA synthesis kit (Marligen, USA). The cDNA was stored at -20°C for subsequent analysis. The primer sequences were all obtained from previous published journal articles [35-37] (Geneworks, Australia) (Table 5). Real-time PCR was performed using the iQ5 multicolour real-time PCR detection system (Bio-Rad, USA), with PCR reactions carried out using the iQ SYBR Green Supermix (Bio-Rad, USA). Fluorescent emission data were captured and mRNA levels were analysed using the critical threshold (*C*T) value. The relative expression of the gene of interest was calculated using the expression 2^-Δ*CT *^and normalised to the cDNA concentration using the Quant-iT OliGreen ssDNA quantitation reagent kit (Invitrogen, Australia) according to the manufactures instructions, and reported as arbitrary units [38].

**Table 5:** Real-time PCR primer sequences for genes of interest

Gene	Forward primer (5'-3')	Reverse primer (5'-3')
NOX 2	TCA AGT GTC CCC AGG TAT CC	CTT CAC TGGCTGTACCAAAGG
p22*^phox^*	GCT CAT CTG TCT GCT GGA GTA	ACGACCTCATCTGTCACTGGA
CuZn-SOD	TGTGTCCATTGAAGATCGTGTGA	TCTTGTTTCTCGTGGACCACC
Mn-SOD	TTAACGCGCAGATCATGCA	CCTCGGTGACGTTCAGATTGT
Ec-SOD	GGCCCAGCTCCAGACTTGA	CTCAGGTCCCCGAACTCATG

The primers were all obtained from previous published sequences and were ordered through Geneworks (Australia): NOX2 [37], p22^phox ^[35], CuZn-SOD, MnSOD and Ec-SOD [36].

### Statistical analysis

Statistical analysis was performed using the SPSS statistical package (version 17.0, SPSS Inc.) for repeated measures ANOVA and one-way ANOVA. The results are represented as mean ± SEM. Comparisons between groups for animal body weight, food intake and water intake data were analysed using repeated measures ANOVA. A post hoc pair-wise comparison was also carried out. Significance was established at the 95% confidence level (*P *< 0.05).

## Competing interests

The authors declare that they have no competing interests.

## Authors' contributions

AP participated in the design of the study, carried out the analysis and interpretation of data and drafted the manuscript. XC helped with the MDA analysis. LL contributed to the interpretation of data and revised the manuscript. PL participated in the design of the study, contributed to the interpretation of data and revised the manuscript. All authors read and approved the final manuscript.
